# A Case of Sarcoidosis with Unusual Radiographic Findings that Developed 5 Years after Silicone Augmentation Mammoplasty Complicated by Miliary Tuberculosis during Corticosteroid Treatment

**DOI:** 10.1155/2011/268620

**Published:** 2011-09-15

**Authors:** Tomoko Miyashita, Katsunobu Yoshioka, Tomoyuki Nakamura, Keiko Yamagami

**Affiliations:** ^1^Department of Internal Medicine, Osaka City General Hospital, Osaka 534-0021, Japan; ^2^Department of Internal Medicine, Osaka City Sumiyoshi Hospital, Osaka 559-0012, Japan

## Abstract

A 54-year-old woman with a past history of silicone augmentation mammoplasty was admitted with fever and dyspnea with diffuse interstitial shadows on computed tomography (CT). Although radiological findings were atypical, we diagnosed sarcoidosis by laboratory, microbiological, and bronchoalveolar lavage fluid analysis. Corticosteroids ameliorated the condition, but she had recurrent of fever and CT revealed miliary nodules while interstitial shadows disappeared. Liver biopsy showed that noncaseating granuloma and Ziehl-Neelsen stain was positive. We diagnosed miliary tuberculosis which developed during corticosteroid therapy. Antituberculotic therapy resulted in favorable outcome. Possibility exists that onset of sarcoidosis was induced by mammoplasty, namely, human adjuvant disease.

## 1. Introduction

Sarcoidosis is a systemic disease of unknown etiology that primarily affects the lungs and is characterized by noncaseous granuloma. On the other hand, tuberculosis is an infectious disease characterized by caseous granuloma. Differentiation of sarcoidosis from tuberculosis is important, as the treatment of sarcoidosis is opposite to that of tuberculosis. However, differentiation can be difficult because clinical and histological findings are similar. Given these similarities, etiological relationships between sarcoidosis and tuberculosis have been documented. The prevalence of sarcoidosis is high in patients who have previously suffered from tuberculosis [1–3], and tuberculosis can develop as an opportunistic infection in patients receiving corticosteroids for sarcoidosis. Sarcoidosis and tuberculosis can even occur simultaneously [4, 5]. 

Human adjuvant disease (HAD) is characterized by autoimmune-like symptoms following cosmetic mammoplasty [6]. Sarcoidosis is one such disease, and some cases have shown development of nervous sarcoidosis [7], pulmonary and skin sarcoidosis [8], and Löfgren syndrome following mammoplasty [9]. 

Herein, we report the case of a patient with sarcoidosis following mammoplasty complicated by miliary tuberculosis during treatment with corticosteroids. Relationships between sarcoidosis, tuberculosis, and HAD are discussed.

## 2. Case Report

A 54-year-old woman was admitted to our hospital with fever, general malaise, and dyspnea. She had undergone bilateral breast augmentation surgery with silicone bags 5 years previously, with resurgery on the right side due to bag rupture 7 months before presentation. Seven days before admission, she became febrile and noticed dyspnea. She visited her family doctor and was diagnosed with pneumonia, but treatment with antibiotic therapy had no noticeable effect. She was then referred to our hospital for further examination and treatment.

The patient was 158 cm tall, weighed 46 kg, and blood pressure was 100/50 mmHg, heart rate was 74 beats/min, body temperature was 37.0°C, and respiratory rate was 30 breaths/min on admission. Inspiratory rales were heard at both lung bases. 

**Figure 1:**
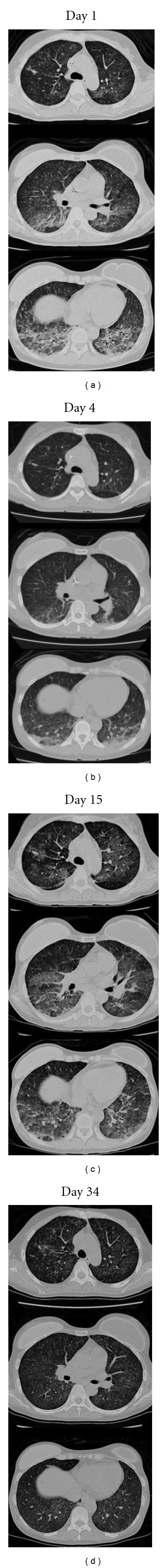
CT findings. (a) On admission, nodular and macular shadows in the right upper and intermediate lobes and left inferior lobe and ground-glass opacities in both inferior lobes were seen. (b) On day 4, interstitial opacity improved. (c) On day 15, interstitial opacity was worsened. (d) On day 34, miliary nodules were seen in both lung fields, but interstitial shadows were ameliorated.

The examination of the blood revealed severe thrombocytopenia with prolonged prothrombin time and high levels of fibrinogen degradation product (FDP) (Table 1). Chemical analysis revealed severe abnormal liver function with jaundice, mild renal insufficiency, and high C-reactive protein (CRP) level. Arterial blood gas levels on room air revealed hypoxemia with hypocapnia. All testing for autoantibodies yielded negative results. Blood culture was negative for bacteria. Computed tomography (CT) of the chest revealed nodular and macular shadowing in the right upper and middle lobes and left inferior lobe and ground-glass opacities in both inferior lobes (Figure 1(a)). Our patient saw an ophthalmologist, and there were no findings suggestive of uveitis.

**Figure 2:**
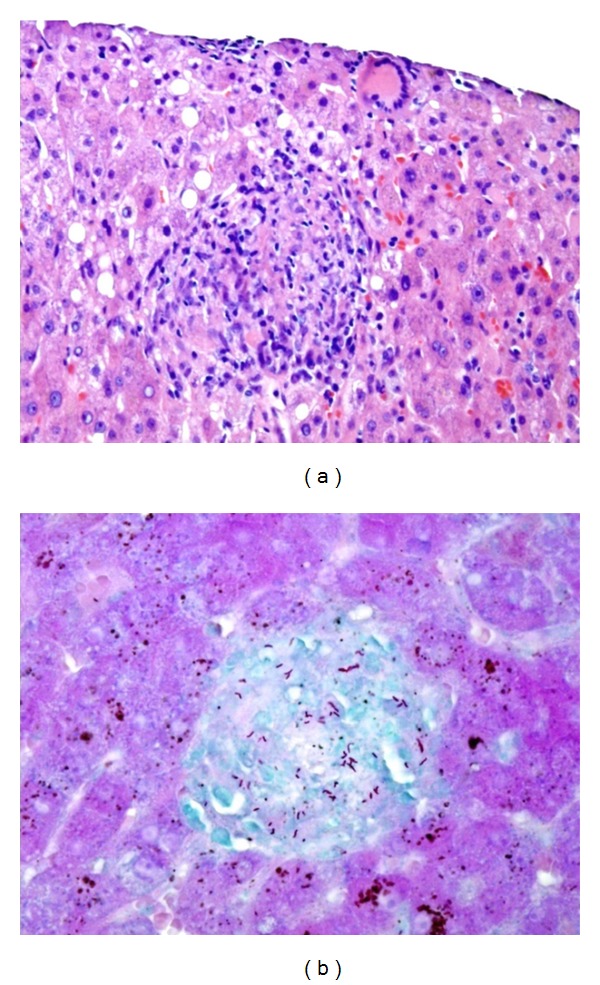
Pathological findings for the liver. (a) Hematoxylin and eosin (HE) staining showing Langhans' giant cell and noncaseating granuloma (×400). (b) Positive results for Ziehl-Neelsen staining (×400).

**Figure 3:**
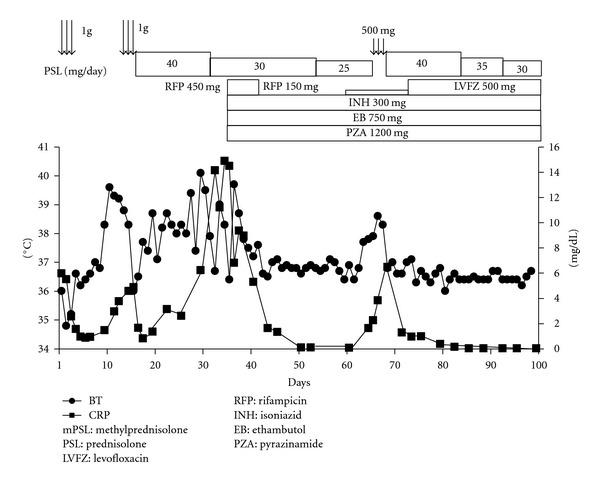
Clinical course after admission.

**Figure 4:**
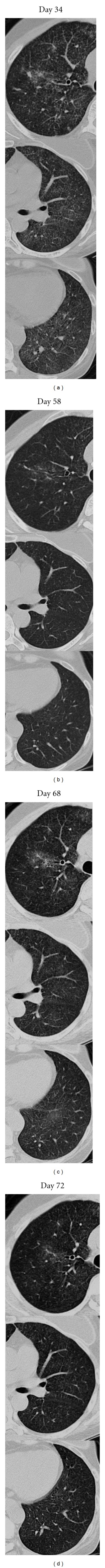
CT findings. (a) On day 34, miliary nodules were seen in both lung fields, but interstitial shadows were ameliorated. (b) On day 58, miliary nodules had almost disappeared from both lung fields. (c) On day 68, new shadowing appeared in both upper lobes. (d) Sickly shadowing had disappeared by day 72.

We diagnosed interstitial pneumonia with hepatic dysfunction and disseminated intravascular coagulation (DIC) and initiated methylprednisolone pulse therapy (1 g/day for 3 consecutive days) for interstitial pneumonia and gabexate mesilate and antithrombin III for DIC. Antibiotic (Meropenem trihydrate 1 g/day, Azithromycin hydrate 500 mg/day) was administrated to cover the possibility of concomitant bacterial pneumonia. On day 4, respiratory status and interstitial opacity on CT were improved (Figure 1(b)). However, fever recurred on day 10 and respiratory status and CT findings were deteriorated on day 15 (Figure 1(c)). Methylprednisolone (1 g/day for 3 consecutive days) was again administered, followed by prednisolone (40 mg/day) to avoid recurring exacerbation of interstitial pneumonia. On day 18, bronchoscopy was performed to identify the cause of interstitial pneumonia. Transbronchial lung biopsy specimens revealed small cell infiltrations of lymphocytes, plasma cells, and neutrophils. Analysis of bronchoalveolar lavage fluid (BALF) revealed an increased number of lymphocytes (52.5%) and a high CD4/CD8 ratio (2.52). However, the cause of interstitial pneumonia remained unclear. Ziehl-Neelsen staining of tissue samples and sputum culture yielded negative results for *Mycobacterium tuberculosis*. Fever recurred on day 20 and liver biopsy was performed to identify the cause of fever. We considered the possibility of sarcoidosis based on elevated levels of angiotensin-converting enzyme (ACE) (63.0 IU/L) and lysozyme (11.8 mg/mL). On day 23, Liver biopsy confirmed Langhans' giant cell and noncaseating granuloma (Figure 2(a)). On day 34, CT was performed to followup the interstitial pneumonia and fever, revealing miliary nodules in both lung fields, typical of miliary tuberculosis, although interstitial shadows had disappeared (Figure 1(d)). On day 35, antituberculosis therapy (isoniazid, 300 mg/day; rifampicin, 450 mg/day; ethambutol, 750 mg/day; pyrazinamide, 1200 mg/day) was applied for miliary tuberculosis after acid-fast bacilli were detected in gastric fluid and sputum, and polymerase chain reaction testing for *Mycobacterium tuberculosis* yielded positive results. Ziehl-Neelsen staining obtained positive results in a liver specimen (Figure 2(b)), but negative results in a lung specimen. Fever subsided following antituberculosis therapy. On day 41, rifampicin was stopped due to leukocytopenia (while blood cell (WBC) count, 1550/mm^3^; neutrophils, 350/mm^3^), likely caused by rifampicin. On day 59, the WBC count was fully recovered, and rifampicin was restarted at a low dose (150 mg). Fever was again seen on day 64, and CT was performed on day 68. Although miliary nodules in both lung fields had almost disappeared (Figure 4(b)), sickly shadowing was newly apparent in both upper lobes (Figure 4(c)). These new lesions were considered highly likely to represent recurrent interstitial pneumonia and not exacerbation of miliary tuberculosis, because exacerbation occurred during treatment for tuberculosis. On day 68, methylprednisolone was restarted at 0.5 g/day for 3 days and the dose of prednisolone was increased to 40 mg/day. Thereafter, fever subsided and the sickly shadowing had disappeared by day 72 (Figure 4(d)). On day 70, we switched from rifampicin to levofloxacin, as leucopenia had again progressed. Prednisolone was gradually tapered to 30 mg/day, and the patient was discharged without recurrence of the disease on day 100 (Figure 3).

## 3. Discussion

The most characteristic feature of the present case was the coexistence of *Mycobacterium tuberculosis* as proven by Ziehl-Neelsen staining and noncaseous granuloma in the liver. Since noncaseous granuloma is not specific for sarcoidosis and can also occur in tuberculosis, the series of events in the present case may be attributable to miliary tuberculosis from the beginning. However, this possibility is considered unlikely for the following reasons.

First, interstitial shadowing on CT and the respiratory status completely ameliorated after first steroid pulse without the use of antituberculotic therapy. The steroid therapy has brought down fever for 7 days. If she was affected by tuberculosis from the beginning, we could not control her fever only by steroid therapy. Second, bronchoscopy on day 18 failed to identify *Mycobacterium tuberculosis* on Ziehl-Neelsen-stained tissue or sputum culture. Although the yield of acid-fast smears from sputum is usually low (46.4–50%) [10], tissue diagnosis is useful, and diagnostic rates of miliary tuberculosis from lung biopsy reache as high as 30–86% [11]. Third, ACE activity can increase in patients with miliary tuberculosis, but decreased in this case after starting corticosteroids without using antituberculotic therapy (pretreatment, 63.0 IU/L; posttreatment, 31.1 IU/L). Fourth, sickly shadowing appeared in both upper lobes while the patient was receiving antituberculotic therapy and was ameliorated by methylprednisolone pulse therapy, suggesting the presence of a pathological condition other than tuberculosis. Taken together, it is reasonable to consider that overt tuberculosis developed during treatment with corticosteroid and another pathological condition existed at the beginning of the clinical course. 

The classic findings of sarcoidosis on lung CT are widespread small nodules with bronchovascular and subpleural distributions, thickened interlobular septa, architectural distortion, and conglomerate masses [12]. Radiographic findings in the present case were thus atypical for sarcoidosis. We, therefore, performed bronchoscopic examination to identify the cause of interstitial pneumonia. Although the specimen obtained by bronchoscopy revealed no specific findings, analysis of BALF revealed that 52.5% lymphocytes and CD4/CD8 ratio was elevated to 2.53, compatible with the diagnosis of sarcoidosis. We, therefore, diagnosed sarcoidosis-associated interstitial pneumonia based on the high level of serum ACE activity, noncaseating granuloma in the liver, and the results of bronchoscopic examination.

The etiology of sarcoidosis remains unclear. However, onset of sarcoidosis in some cases is causally related to mammoplasty [7–9]. In the present case, a causal relationship between onset of sarcoidosis and HAD was suggested by the fact that the patient had a past history of bilateral mammoplasty with silicone bags 5 years previously and a bag had ruptured 7 months before presentation, followed by development of sarcoidosis. Miyoshi et al. originally coined the term HAD in 1964 [13]. Their report described cases of autoimmune diseases in two Japanese women who had previously undergone mammoplasty but failed to meet the criteria for autoimmune diagnoses including scleroderma, progressive systemic sclerosis, systemic lupus erythematosus, rheumatoid arthritis, or Sjögren's syndrome [6]. Interstitial pneumonia is rare as a symptom of HAD. However, a case of HAD-associated polymyositis, Sjögren syndrome, and interstitial pneumonia that developed 30 years after mammoplasty has been reported [14]. HAD may thus have induced or triggered the onset of sarcoidosis in the present case. Until now, majority of the patients who have removed their silicone bags have had dramatic improvement. However there were also some reports that the symptoms of HAD have not improved after the removal of silicone bags. In this case, we did not remove the silicone bags because the benefit of disposal of silicone bags has not been proven [15]. 

In summary, we reported the case of a patient with sarcoidosis following mammoplasty complicated by miliary tuberculosis during treatment with corticosteroids. HAD may have induced or triggered the onset of sarcoidosis. Sarcoidosis needs to be distinguished from miliary tuberculosis because the therapeutic strategies for these two pathologies are in opposition.

## Figures and Tables

**Table 1:** Laboratory data on admission.

Blood cell count	
WBC	7780/mm3
Neutrophils	86.7%
Lymphocytes	12.3%
Monocytes	0.9%
Eosinophils	0.0%
Basophils	0.1%
RBC	424 × 104/mm3
Hb	13.1 g/dL
Ht	35.0%
Plt	2.1 × 104/mL

Blood gas analysis	
pH	7.467
pCO_2_	26.2 mmHg
pO_2_	69.2 mmHg
HCO_3_	18.7 mmol/L
BE	−3.3 mmol/L
SAT	93.8%
Lactic acid	20 mg/dL

Blood chemistry	
TP	4.8 g/dL
Alb	2.4 g/dL
T-Bil	6.8 mg/dL
D-Bil	4.8 mg/dL
AST	332 IU/L
ALT	185 IU/L
LDH	963 IU/L
ALP	560 IU/L
ChE	198 IU/L
BUN	51.1 mg/dL
Cre	1.81 mg/dL
CRP	5.97 mg/dL
CK	150 IU/L
Na	132 mEq/L
K	3.0 mEq/L
Cl	102 mEq/L
SP-D	429 ng/mL
KL-6	674 U/mL
ACE	63.0 IU/L
Lysozyme	11.8 mg/mL

Serological test	
HBsAg	(—)
Anti-HBc-IgM Ab	(—)
Anti-HCV Ab	(—)
Anti-HA-IgM Ab	(—)
RF	(—)
ANA	<×40
Myeloperoxidase ANCA	<3.5 U/mL
Proteinase-3 ANCA	<1.4 U/mL
Anti-Smith antibody	(—)
AMA	(—)
Anti-Scl antibody	(—)
Anti-RNP antibody	(—)

Coagulation studies	
PT-INR	2.23
FDP	101.9 mg/mL
Fbg	<50 mg/dL
AT-III	15%

AST: aspartate aminotransferase, ALT: alanine aminotransferase, LDH: lactate dehydrogenase, ALP: alkaline phosphatase, CRP: C-reactive protein, RF: rheumatoid factor, ANA: antinuclear antibody, ANCA: antineutrophil cytoplasmic antibody, AMA: antimitochondrial antibody, Scl: Scleroderma, RNP: ribonucleoprotein, Plt: platelet, SAT: saturation, TP: total protein, SP-D: pulmonary surfactant protein D, KL-6: sialylated carbohydrate antigen.
